# ROCK Inhibitors in Corneal Diseases and Glaucoma—A Comprehensive Review of These Emerging Drugs

**DOI:** 10.3390/jcm12216736

**Published:** 2023-10-25

**Authors:** Luca Pagano, Jason William Lee, Matteo Posarelli, Giuseppe Giannaccare, Stephen Kaye, Alfredo Borgia

**Affiliations:** 1 Department of Biomedical Sciences, Humanitas University, 20072 Milano, Italy; luca.pagano@humanitas.it; 2 Clinical Eye Research Centre, St Paul’s Eye Unit, Royal Liverpool University Hospital, Liverpool L7 8YE, UK; jason.lee1@rlbuht.nhs.uk; 3 Department of Corneal Diseases, St. Paul’s Eye Unit, Royal Liverpool University Hospital, Liverpool L7 8YE, UK; mposarelli@gmail.com (M.P.); stephen.kaye@liverpoolft.nhs.uk (S.K.); alfredo.borgia@liverpoolft.nhs.uk (A.B.); 4 Ophthalmology Unit of the Department of Medicine, Surgery and Neuroscience, University of Siena, 53100 Siena, Italy; 5 Eye Clinic, Department of Surgical Sciences, University of Cagliari, 09124 Cagliari, Italy; 6 Eye Unit, Humanitas-Gradenigo Hospital, 10122 Turin, Italy

**Keywords:** rho kinase, cornea guttata, Descemet membrane, glaucoma, ripasudil, netarsudil

## Abstract

Rho kinase (ROCK) inhibitors have gained significant attention as emerging novel treatment options in the field of ophthalmology in recent years. The evidence supporting their efficacy in glaucoma and corneal pathology includes both in vitro and clinical studies. Among the available options, ripasudil and netarsudil have emerged as the leading ROCK inhibitors, and some countries have approved these therapeutic options as treatments for glaucoma. Various dosing regimens have been studied, including monotherapy and combination therapy, especially for patients with secondary glaucoma who are already on multiple medications. Another rising application of ROCK inhibitors includes their use as an adjunct in surgical procedures such as Descemetorhexis Without Endothelial Keratoplasty (DWEK), Descemet Stripping Only (DSO) to accelerate visual recovery, glaucoma surgeries to reduce scarring process and allow better intraocular pressure (IOP) control, or after complicated anterior segment surgery to treat corneal oedema. This article provides a comprehensive overview of the existing literature in the field, offering recommendations for prescribing ROCK inhibitors and also discussing patient selection, drug efficacy, and possible adverse effects.

## 1. Introduction

Addressing glaucoma and corneal disease can present substantial difficulties, often necessitating medical therapy as the primary mode of treatment. Polypharmacy is prevalent among patients with glaucoma; half of patients with newly diagnosed glaucoma initially require more than one medication to control IOP, and a further 30% require add-on treatment after one year [1]. Furthermore, many of the current medications have systemic side effects and there is evidence suggesting that intensification of medical therapy has diminishing returns and increased clinical and economic burdens [2]. There is, therefore, an ongoing need for novel therapies, and one promising emerging therapy is Rho kinase (ROCK) inhibitors, which have gained significant attention in the last ten years. The Rho kinase (ROCK) signaling cascade is ubiquitously present in all tissues of the human body, and it regulates different cellular processes such as replication, proliferation, and apoptosis [3]. In the eye, it regulates the physiological properties of the trabecular meshwork and corneal endothelium. Thus, the ROCK signaling cascade was hypothesized to be a potential therapeutic target. In addition to opening new therapeutic options in glaucoma, ROCK inhibitors have shown promise in the treatment of corneal endothelial diseases by promoting corneal endothelial cell regeneration and both functional and morphological recovery [4]. Laboratory studies have suggested that ROCK inhibitors improve cellular attachment and proliferation of cultured endothelial cells in vitro and improve wound healing on ex vivo corneas [5]. They facilitate cell cycle progression from the G1 to S phase and prevent actinomyosin contraction by inhibiting the Rho kinase signaling cascade [6]. Therefore, ROCK inhibitors serve a niche in either in current routinely performed anterior segment surgery to minimize complications or to enhance novel techniques such as Descemetorhexis Without Endothelial Keratoplasty (DWEK) [7]. However, there are variable levels of acceptance among healthcare professionals worldwide, and these drugs are still far from being routinely used in routine ophthalmology practice.

Three commercially available ROCK inhibitors currently exist: ripasudil (Glanatec^®^), netarsudil (Rhopressa^®^), and fasudil. Ripasudil has been used in the treatment of ocular hypertension and glaucoma in Japan since September 2014 and has been recently approved for commercial use in the United Kingdom. Netarsudil has been used for glaucoma treatment in the United States since late 2017 and more recently has been approved in Europe in November 2021. Fasudil was originally approved in Japan in 1995 for the treatment of cerebral vasospasms caused by subarachnoid hemorrhage [8], and it is used in ophthalmology for the treatment of diabetic macular edema (DME) [9]. Finally, two ROCK inhibitors have not been approved yet: SNJ-1656, currently in phase II trial for controlling intraocular pressure (IOP) [10], and Y-27632, still in a pre-clinical phase for use in corneal endothelial diseases [5]. 

As the utilization of ROCK inhibitors are increasing among ophthalmologists globally, it is crucial to better understand the potential benefits and safety of these pharmaceutical products for both approved and off-label use. In this regard, we present a comprehensive review covering the current level of knowledge in the existing literature, aiming to provide evidence-based recommendations for prescribing ROCK inhibitors. This review will also discuss patient selection, efficacy, and adverse effects of these drugs with a particular focus on the two FDA-approved molecules ripasudil and netarsudil. 

## 2. The Rho Kinase Pathway

**Figure 1:**
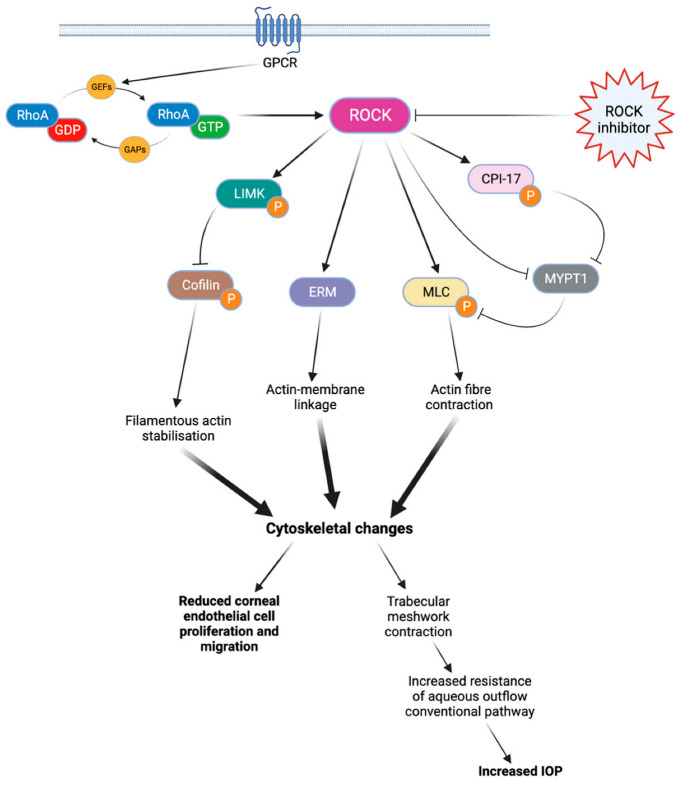
Rho kinase cascade of activation. When coupled with GTP, RhoA protein can phosphorylate various target proteins and ultimately lead to cytoskeletal changes. These cytoskeletal modifications limit endothelial cell proliferation and migration, as well as aqueous outflow through the conventional pathway. By blocking this cascade, ROCK inhibitors can improve corneal endothelial cell migration and reduce the IOP. ROCK: Rho kinase. LIMK: LIM kinase. ERM: ezrin-radixin-moesin. MLC: myosin light chain. CPI-17: C-kinase-potentiated protein phosphatase 1 inhibitor of 17 kDa. MYPT1: Myosin phosphatase target subunit 1. IOP: intraocular pressure.

There are two isoforms of ROCK, ROCK1 (ROKβ) and ROCK2 (ROKα), which may have slightly different effects depending on the isoform [11,12]. The activation of RhoA is regulated by guanine nucleotide exchange factors (GEFs), GTPase activating proteins (GAPs), and guanine nucleotide dissociation inhibitors (GDIs) [11,12]. Once activated, RhoA proteins trigger the activation of ROCKs, which phosphorylate various target proteins involved in actin-related processes such as actomyosin contraction, cell adhesion, cell morphology and stiffness, and cell migration [11]. These general processes play a key role in modulating various eye mechanisms (Figure 1), such as aqueous humor outflow regulation [11,13], corneal tissue regeneration [13], or optic nerve vessel vasodilation [13]. 

In glaucoma, endothelin-1 (ET-1)-induced ROCK signaling plays a role in regulating retinal blood flow and vasomotor tone, resulting in the promotion of vasoconstriction [14]. Administration of ROCK inhibitors has been shown to enhance ocular blood flow in both normal and disease rabbit models, as well as in normal rat models. This was demonstrated by increased blood flow in the optic nerve head when assessed using LASER speckle flowgraphy [8,15]. ROCK inhibitors administration induces the relaxation of vascular smooth muscle cells, leading to the dilation of ONH blood vessels. 

Additionally, ROCK inhibitors can alter the trabecular meshwork cell shapes improving the control of intraocular pressure (IOP). In the cornea, ROCK inhibition has been shown to induce metabolic changes in endothelial cells, such as increased mitochondrial metabolic activity and upregulation of oxidative phosphorylation through the AMPK pathway [16], which would support the change in cellular function described above. Furthermore, the ROCK pathway has also been implicated in retinal diseases such as diabetic retinopathy [17] and diabetic macular oedema [18]. 

## 3. Evidence for Clinical Efficacy and Safety of Ripasudi

### 3.1. Ripasudil in Glaucoma

In Phase 1 and Phase 2 trials, ripasudil has demonstrated a significant reduction in IOP when used as monotherapy in patients with open-angle glaucoma (POAG) or ocular hypertension (OHT) [19,20]. In particular, a dose-dependent reduction in the IOP was observed with ripasudil 0.1–0.4% twice daily versus placebo over 8 weeks. Further, Tanihara et al. investigated the use of 0.4% ripasudil as a single therapy, or in addition to either latanoprost or timolol, in POAG and OHT [21]. In these trials, ripasudil demonstrated a significantly greater IOP reduction over 8 weeks at trough and peak levels when combined with prostaglandins or beta-blockers as compared to placebo. Furthermore, the authors confirmed these results in a multicentric, prospective 52-week study that enrolled 388 patients with POAG, OHT, and exfoliation glaucoma [22]. In this study, authors divided the patients into four cohorts: cohort 1, treated with 0.4% ripasudil as monotherapy; and cohorts 2 to 4, treated with ripasudil and prostaglandins, beta-blockers, and fixed-association prostaglandins-beta-blockers, respectively. Ripasudil showed significant IOP reduction when utilized in monotherapy and additive therapy. Interestingly, 94.1% of subjects experienced adverse events, in particular conjunctiva hyperemia (75%), blepharitis (21%), allergic conjunctivitis (17%), eye irritation (10%), conjunctivitis (7%), and eyelids pruritus (4.5%). In most of the patients the conjunctival hyperemia was mild and resolved spontaneously, whereas 22% of subjects required further treatment. Interestingly, the brush cytology did not show a correlation between the presence of eosinophils and an allergic reaction. Finally, they also evaluated the effectiveness of ripasudil in a larger post-marketing observational study over 3 months, with a sample size of over 3000 participants [23]. All groups, including ripasudil as newly initiated monotherapy, combination therapy, or switched therapy, showed a significant reduction in mean IOP over the study period in multiple glaucoma subtypes including exfoliation and uveitic glaucoma. In terms of safety, only 8% of patients experience adverse drug reactions (ADRs), with the most common side effects represented by conjunctival and ocular hyperemia. The overall IOP mean reduction was −2.6 ± 4.1 mmHg compared to the baseline, with the highest reduction observed in subjects with NTG (−3.9 ± 5.3 mmHg). Within patients with secondary glaucoma, good efficacy was observed in cases of exfoliation glaucoma (−3.0 ± 5.5 mmHg), uveitis-associated glaucoma (−4.7 ± 7.2 mmHg), and steroid glaucoma (−5.5 ± 6.0 mmHg), whereas a non-significant IOP reduction was found in subjects with neovascular glaucoma (−2.8 ± 12.1 mmHg, *p* = 0.669). 

Additionally, various case series and observational studies [24,25,26,27] have shown the effectiveness of ripasudil as an adjunct or monotherapy in specific subtypes of glaucoma such as exfoliation and uveitic glaucoma, and in patients where maximum medical therapy failed to control IOP adequately. In a study with 30 participants, the combination of ripasudil and prostaglandin analogs showed a significant additional reduction in intraocular pressure compared to prostaglandin analog monotherapy in patients with normal tension glaucoma [28]. The combination treatment resulted in significant IOP lowering at 1 month and 3 months. Jethva et al. conducted a small prospective study in patients with inadequately controlled IOP on two or more treatments [29]; ripasudil was added to patients’ ongoing glaucoma therapy, and the authors observed a significant reduction in IOP at 3 months. Furthermore, Tanihara et al. found that ripasudil–brimonidine fixed-dose combination therapy was more effective than monotherapy of either drug and just as effective as administering ripasudil shortly followed by brimonidine, but with the added benefit of potentially improving patients’ compliance [9]. 

Ripasudil has also been studied as an adjunct to surgical glaucoma procedures such as trabeculectomy [30]. Mimura et al. investigated the potential application of ripasudil in lowering IOP after trabeculectomy for patients with uveitic glaucoma, who often have poorer outcomes post-surgery. They found that ripasudil could reduce the need for bleb needling or revision, and all participants in the ripasudil treatment group had a significant reduction in IOP compared to the control group at 3 months post-operatively. Further trials are currently investigating the use of ripasudil drops after bleb needling without antimetabolite agents [31]. In selected cases, ripasudil treatment after bleb needle revision could suppress the fibrotic processes and improve the bleb filtration, reducing the need for antimetabolite injections. 

To date, no studies have compared ripasudil as a monotherapy to first-line glaucoma drugs such as prostaglandins and beta-blockers. However, ripasudil is demonstrating promising results in reducing the IOP when used as a monotherapy or in combination with other agents or procedures. More extensive studies are needed to establish its efficacy as a standalone treatment and further explore its potential in different types of glaucoma.

### 3.2. Ripasudil in Corneal Diseases

Descemet’s membrane removal in procedures like Descemetorhexis Without Endothelial Keratoplasty (DWEK) or Descemet’s Stripping Only (DSO) for Fuchs Endothelial Corneal Dystrophy (FECD) has been shown to improve corneal clarity and vision and to concurrently reduced the need for corneal graft procedures [7]. In this scenario, the use of ripasudil as an adjunct therapy to these procedures has shown promising results [32,33]. Multiple studies have highlighted the potential of ripasudil in achieving corneal clearance and improving visual outcomes in DWEK/DSO procedures. A recent meta-analysis of 68 patients undergoing DWEK found that faster corneal clearance was achieved in patients treated with a ROCK inhibitor as compared to non-treated subjects (4.9 weeks vs. 10.1 weeks, respectively, *p* < 0.001) [34]. A follow-up study in 2021 further supported the use of ripasudil, with corneal clearance observed in the majority of the cases [35]. Macsai et al. compared DWEK combined with ripasudil to standalone DWEK [36]. Eighteen subjects were included; nine were assigned to the observation group (DWEK only) and nine to the treatment group (DWEK plus netarsudil). In the ripasudil group, patients experienced a faster visual recovery (4.6 vs. 6.5 weeks, *p* < 0.01) and a higher average at 3 months (859 vs. 552, *p* < 0.01), 6 months (934 vs. 672, *p* < 0.01), and 12 months (1086, vs. 736, *p* < 0.01) as compared to the DWEK-only group. Further, peripheral ECD did not significantly change from baseline to 12 months post-operatively (1239 vs. 1233 cells/mm^2^, *p* < 0.1) in subjects treated with netarsudil, whereas the observation group had a statistically significant reduction in ECD (1257 vs. 1142 cells/mm^2^, *p* < 0.01). Overall, studies have shown patients treated with DWEK/DSO require a longer time to achieve a visual outcome similar to those treated with Descemet’s Membrane Endothelial Keratoplasty (DMEK) [33]. Therefore, the delay in visual recovery may give ripasudil a valuable role, considering that DWEK/DSO procedures do not require donor corneal tissue and could help overcome the problem of limited availability of corneal tissues [37]. Due to its healing effect on corneal endothelium, other applications of ripasudil have been studied. Cataract or anterior segment surgeries are known to be associated with a risk of postoperative corneal oedema due to endothelial cell damage. Studies have shown that administering ripasudil in the post-operative period has a protective effect on endothelial cell density [38], and may improve visual recovery after complicated procedures [39,40]. In both Descemet’s Stripping Automated Endothelial Keratoplasty (DSAEK) and penetrating keratoplasty corneal grafts that failed to clear with conservative management, vision can be rescued with ripasudil therapy [41]. Ripasudil also shows promise in other pathology which can result in oedema such as acute hydrops in keratoconus [42]. Eslami et al. presented the case of a 32-year-old male diagnosed with corneal hydrops. The patient was started on topical netarsudil 0.4% twice a day. At 3 weeks of follow-up, VA was slightly better and corneal oedema had resolved completely. The patient underwent corneal lamellar transplant successfully, and at 14 months the BCVA was 0.18 logMAR. In this case, the use of netarsudil allowed the treatment of corneal oedema and improved the view during the corneal transplant. 

### 3.3. Safety Profile of Ripasudil

The adverse effects of ripasudil have been well studied (Table 1). The most significant adverse drug reactions (ADR) reported at one year by Tanihara et al. were conjunctival hyperemia (74.6%) and blepharitis (20.6%) [22]. However, the majority of hyperemia cases were only classed as ‘mild’ in severity and usually resolved within 2 h [43]. The main reasons for discontinuing ripasudil were usually blepharitis and symptoms such as pruritis and eyelid redness, rather than conjunctival hyperemia [44]. There have been some case reports of a further adverse effect of honeycomb/reticular epithelial oedema [35,45,46] not reported in previously discussed larger clinical trials. This effect is usually transient, but in one case the patient required a repeat corneal graft procedure to improve visual function [45]. 

Tanihara et al. later followed up POAG and OHT patients over 12 months and then 24 months [47,48] and demonstrated an acceptable safety profile of twice daily ripasudil. Out of 3374 participants, the rate of adverse events was 25.3%, of which 8.6% was accountable for blepharitis (the most common adverse effect). This was only slightly elevated compared to the results at 1 year, and 87% of participants recovered from the adverse events. No serious adverse events were reported at 24 months. Ripasudil-associated blepharitis was significantly correlated with a past medical history of atopy or drug allergy. However, ripasudil is still considered safe to be used in those with a sulfonamide antibiotic allergy, as there is no evidence of cross-reactivity in the current literature [49].

## 4. Evidence for Clinical Efficacy and Safety of Netarsudil

### 4.1. Netarsudil in Glaucoma

Netarsudil has been shown to lower the IOP by improving the outflow facility and reducing the episcleral venous pressure (EVP) [50]. In this multicenter, randomized, placebo (vehicle)-controlled, double-masked Phase 2 study, authors included 20 patients, and eyes were randomized to be treated with placebo or netarsudil. For each subject, one eye received one drop of netarsudil 0.02%, and the fellow eye received one drop of vehicle once a day in the morning for 7 days. The primary endpoint was the change in mean diurnal trabecular outflow facility compared to baseline, and the secondary objectives were the differences in IOP and episcleral vein pressure changes between drug and placebo, as well as ocular and systemic safety. At day 8, a significant difference in diurnal outflow facility was observed in the netarsudil group as compared to the placebo (0.039 ± 0.040 µL/min/mmHg vs. 0.007 ± 0.028, *p* < 0.01). Further, IOP changes from baseline were significantly higher in the eye treated with netarsudil compared to the fellow eye (−4.52 ± 1.58 mm Hg vs. −0.98 ± 1.60 mm Hg, *p* < 0.01). Interestingly, the EVP decreased in the drug group compared to baseline (−9.5%, *p* < 0.01) and increased in the vehicle group (3.1%, *p* = 0.81), with a between-treatment difference of −12.6% (*p* < 0.001 vs. vehicle). These results suggest that netarsudil could influence the distal portion of the conventional outflow pathway beyond Schlemm’s canal. 

One of the first clinical studies by Bacharach et al. compared netarsudil to latanoprost in patients with POAG or OHT. In this double-masked, parallel comparison study, patients were randomized to receive netarsudil 0.01%, netarsudil 0.02%, or latanoprost 0.005% for 28 days. Subjects with POAG or OHT were included. At days 14 and 28 of treatment, all three groups showed a reduction in IOP as compared to unmedicated baseline (*p* < 0.01). Although netarsudil did not meet the criterion for noninferiority to latanoprost, authors observed that netarsudil 0.02% had similar efficacy in patients with a baseline IOP ≤ 26 mmHg [51]. 

**Figure 2:**
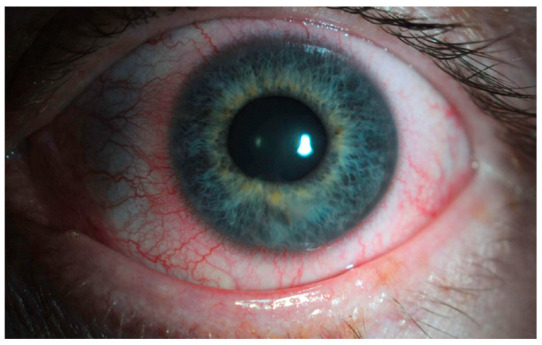
Unilateral mild conjunctival hyperemia (left eye) in a patient treated with latanoprost/netarsudil combination for glaucoma. The patient reported that the redness in the eye typically resolved within two hours following the application of the drops.

The main clinical trials that support the use of netarsudil for glaucoma consist of the ROCKET and MERCURY studies. MERCURY-1 and MERCURY-2 trials demonstrated a significantly higher decrease in IOP with the netarsudil/latanoprost fixed-dose combination compared to either of the therapies individually [52,53]. In MERCURY-3, authors conducted a 6-month prospective, double-masked, randomized, multicenter, active-controlled, parallel-group, non-inferiority study [54]. They included 430 patients from 58 clinical sites of 11 European countries, and subjects were randomized to receive netarsudil/latanoprost 0.02%/0.005% (NET/LAT) or bimatoprost 0.03%/timolol maleate 0.5% (BIM/TIM). For the primary endpoint, NET/LAT FDC demonstrated non-inferiority to BIM/TIM, with a between treatment difference in IOP of ≤1.5 mmHg achieved at all time points and ≤1.0 mmHg at the majority of time points from week 2 through week 12. Interestingly, two time points showed a statistically significant difference in mean IOP: 08:00 at week 6 and week 12 in favor of BIM/TIM. These results are consistent with previous works comparing netarsudil with timolol [55]. The ROCKET trials compared netarsudil to timolol as standalone therapy for reducing IOP. The ROCKET-1 and ROCKET-2 trials showed that netarsudil was as effective as timolol in reducing the IOP in patients with a baseline IOP of <25 mmHg [56]. However, it is important to note that netarsudil use was associated with a greater incidence of adverse events such as conjunctival hyperemia that caused discontinuations of the drug (Figure 2). The ROCKET-4 study [55,57], which had broader inclusion criteria, demonstrated non-inferiority of netarsudil once daily to timolol twice daily in patients with baseline IOP < 30 mmHg. Mathur et al. conducted a real-world, open-label observational study and deemed netarsudil monotherapy to be effective yet safe [58]. 

The effectiveness of netarsudil in specific clinical sub-cohorts, such as secondary glaucoma and patients on maximal tolerated medical therapy, is still being explored. Preliminary evidence suggests that netarsudil can provide additional IOP-lowering effects in patients with uveitic glaucoma on maximal tolerated medical therapy [59]. Netarsudil has also been shown to be effective in a cohort of patients with Sturge-Weber Syndrome on maximal medical therapy by reducing EVP [60], and it demonstrated similar efficacy to latanoprostene bunod when used as adjunct therapy in patients on maximal therapy for POAG [61]. The efficacy of netarsudil has also been compared to ripasudil in the J-ROCKET study and demonstrated a stronger IOP-lowering effect [62]. Like ripasudil, netarsudil has also been studied in the context of glaucoma surgery. Xu et al. investigated the effect of netarsudil on patients who had undergone Kahook blade goniotomy [63], and they observed a greater decrease in IOP as compared to goniotomy-naïve patients. This was thought to be due to netarsudil’s effect on lowering the EVP.

### 4.2. Netarsudil in Corneal Diseases

A recent randomized study investigated the use of netarsudil in patients with symptomatic Fuchs Endothelial Corneal Dystrophy (FECD) [64]. The study included 29 subjects who were either given netarsudil 0.02% once daily or a placebo for three months. The results showed that netarsudil monotherapy led to a significant reduction in central corneal thickness and improvement in best-corrected visual acuity compared to the placebo. Another study by Lindstrom et al. demonstrated significant improvement in central corneal thickness as well as visual acuity and patient-reported FECD-associated symptoms when once-daily dosing was used [65]. 

Netarsudil has not been extensively studied as an adjunct therapy in DWEK/DSO, with only three case reports available. However, these studies showed improved endothelial cell density and resolution of corneal edema in patients treated with netarsudil [66,67,68]. The advantage of netarsudil is its once-daily dosing, which provides a practical benefit over ripasudil and may improve patients’ compliance. Prospective trials are needed to further explore the potential application of netarsudil in corneal endothelial diseases. 

### 4.3. Safety Profile of Netarsudil

The ROCKET [55] and MERCURY [69] trials evaluated the safety profile of netarsudil 0.02% once daily as monotherapy or as a fixed-dose combination with latanoprost 0.005%. In a pooled analysis of safety from the ROCKET trials, no serious ocular adverse events were reported when netarsudil was used as a standalone treatment, and the overall rate of serious ADRs (including non-ocular) was 3.3% for netarsudil-treated patients, similar to the rate of 3.2% in timolol-treated patients [55]. The non-ocular serious events reported for the netarsudil group included coronary artery disease, myocardial infarction, atrial fibrillation, and prostate cancer. The MERCURY-2 trial demonstrated that the most common adverse effect was conjunctival hyperemia, which occurred in 55% of patients using netarsudil as a standalone treatment. This was higher than that observed in standalone treatment with latanoprost (22.3%) and timolol (10.4%), but no patients on netarsudil discontinued the treatment. The majority of the conjunctival hyperemia cases were classified as ‘mild’. The other most common adverse effects included cornea verticillata, which was reported between 9% and 15% of patients with an onset of 2–13 weeks. The corneal appearance was similar to that seen with the use of some systemic medications, most notably amiodarone. It is believed that ROCK inhibitors penetrate the lysosomes within the basal epithelial layer of the cornea; within these lysosomes, they bind to cellular lipids. These complexes of medication and lipids are resistant to enzymatic breakdown and build up as deposits in the cornea [70]. This might have notable implications for individuals with glaucoma experiencing reduced contrast sensitivity due to their underlying optic neuropathy. However, none of these adverse effects had any influence on visual acuity, and they resolved once netarsudil was discontinued.

MERCURY-2 demonstrated that the rate of serious adverse events in the fixed-dose combination group was lower than in either netarsudil or latanoprost monotherapy; none of which were considered to be treatment-related. It is interesting to note that blepharitis is not a common adverse effect of netarsudil in contrast to ripasudil, and the reasons for this remain unclear. As with ripasudil, various studies reported the incidence of honeycomb corneal oedema caused by netarsudil [46,71,72]. However, this adverse effect was not found in any participant in the MERCURY-2 study [53]. The nature of corneal oedema seems to vary between netarsudil and ripasudil, with the onset being faster in netarsudil. Patients that develop corneal oedema are likely to have risk factors such as reduced endothelial cell count, epithelial defects, or a history of penetrating keratoplasty [46,71]. Netarsudil-associated cornea oedema has been reported to occur in children [73]; one case of corneal flattening was also reported in a child [74].

## 5. Future Directions

ROCK inhibitors are showing promising results, but their clinical use is still limited. In future, there is the possibility that more specific molecules will be introduced to selectively target the trabecular meshwork, the corneal endothelium, and the optic nerve. More selective ROCK inhibitors could potentially increase their clinical efficacy and reduce the side effects. Further, new studies are focusing on direct genetic modulation of ROCK signaling to clarify the mechanism of aqueous outflow, as well as to find novel glaucoma gene therapies [75].

In cornea, new less-invasive surgical techniques such as DSAEK and DMEK have allowed the treatment of endothelial diseases with better clinical outcomes as compared to PK. These new procedures have the advantage over PK to offer a faster visual recovery, a better refractive outcome, and a lower rejection risk. However, they still require a learning curve, especially for DMEK surgeries, and they are associated with graft rejection risk. In this scenario, the use of ROCK inhibitors has been proposed for tissue engineering therapies [76]. In particular, the injection of corneal endothelial cells could be enhanced using ROCK inhibitors to improve cell adhesion and replications [76]. Further clinical studies are needed to confirm the safety and efficacy of these new engineering therapies as compared to conventional corneal grafts. Initiating ROCK inhibitors in the early stages of glaucoma can be advantageous as they have the potential to reduce intraocular pressure (IOP) while the trabecular meshwork is functioning properly. This approach is particularly beneficial for patients with steroid-induced ocular hypertension or uveitic glaucoma. However, a comprehensive understanding of the full benefits of ROCK inhibitor therapy in early-stage glaucoma necessitates further investigation.

Although ROCK inhibitors represent an innovative category of topical medications for lowering IOP, it is crucial to conduct more clinical trials and post-marketing studies to establish optimal treatment protocols for glaucoma patients.

Lastly, ROCK inhibitors could also serve in modulating wound healing response following glaucoma filtration surgery. The wound healing process depends on various mechanisms such as cell proliferation and migration, necessitating constant and active changes in the cell’s cytoskeleton.

In vitro studies revealed the role of Rho-ROCK expression in Tenon fibroblasts (TF), which are central to ocular wound healing. Specifically, they have demonstrated that the use of ROCK inhibitors suppresses wound healing activities of TF in vitro. Exposure to ROCK inhibitors, significantly inhibits fibroblast proliferation, adhesion, and contraction [77,78]. Honjo et al. also demonstrated that topical treatment with a ROCK inhibitor effectively reduces subconjunctival scarring at day 7 after experimental glaucoma surgery in rabbits [77]. While long-term experiments on the effect of ROCK inhibition on collagen deposition and bleb survival after glaucoma filtration surgery are still lacking, some data in rabbit models and small groups have shown the inhibition of the proliferation of human TF and the differentiation of fibroblasts into myofibroblasts [79]. As a result, a postoperative topical treatment with a ROCK inhibitor (AMA0526) significantly improved the outcome of glaucoma filtration surgery. Compared to eyes treated with a vehicle, AMA0526 resulted in increased bleb area and prolonged survival. Histological evaluation revealed that blebs treated with the ROCK inhibitor exhibited reduced inflammation, angiogenesis, and collagen deposition at the filtration surgery site [79]. Additionally, experimental evidence suggests that, aside from being a regulator of the cytoskeleton, ROCK also plays a significant role in the inflammatory process [80], with potential benefits of ROCK inhibition in treating conditions like rheumatoid arthritis [81] and Crohn’s disease [82], where it inhibits NF-kb activation and reduces the production of inflammatory cytokines. ROCK inhibitors have demonstrated anti-inflammatory, antiangiogenic, and antifibrotic effects in various animal models, including those for ocular conditions like corneal wound healing and age-related macular degeneration. Consequently, targeting the Rho-ROCK pathway offers promise for modulating the wound healing response following glaucoma surgery.

## 6. Conclusions

ROCK inhibitors such as ripasudil and netarsudil have shown promise as safe, emerging treatment options across different sub-specialties of ophthalmology. In glaucoma, they have shown efficacy as monotherapy and open new avenues for treatment for patients who have inadequately controlled IOP on maximum medical therapy. By acting on the dysfunctional trabecular meshwork, these agents address the underlying cause of glaucoma, as opposed to other agents like beta-blockers that only reduce aqueous humor secretion. In both glaucoma and corneas, ROCK inhibitors have also proven to be an effective adjunct to surgery, such as trabeculectomy or Descemetorhexis Without Endothelial Keratoplasty. Combination with other pre-existing medications such as brimonidine has shown to have further additive effects compared to monotherapy. For now, it remains unclear whether ripasudil or netarsudil is superior. Only one study so far (J-ROCKET) has compared ripasudil versus netarsudil and concluded that netarsudil had superior IOP-lowering effects with fewer side effects; however, further studies would be needed to confirm this observation as the number of cases of reticular epithelial oedema appears to be higher with netarsudil use.

## Figures and Tables

**Table 1:** List of similarities and difference between ripasudil and netarsudil.

Similarities	Differences
Overall safety profiles acceptable for clinical use.	The adverse drug reaction (ADR) rate for netarsudil was 3.3% (RCT) versus 18.7% for ripasudil (post-marketing surveillance).
Conjunctival hyperemia is the most frequent adverse event.	Blepharitis is the most common reason for discontinuation of ripasudil treatment but is not a prominent side effect of netarsudil.
	The incidence of severe conjunctival hyperemia is greater in ripasudil compared to netarsudil.
	Netarsudil is associated with cornea verticillata as an adverse drug reaction, whereas this has not been observed with ripasudil.
Reticular honeycomb epithelial edema has been observed with both drugs.	Reticular honeycomb epithelial oedema seems to be more frequent with netarsudil, and it has not been observed in the randomized controlled trials for ripasudil.

## Data Availability

Not applicable.
